# Supplementary material for: Microbiota-driven interleukin-17-producing cells and eosinophils synergize to accelerate multiple myeloma progression
Source: Nat Commun. 2018 Dec 3;9:4832. doi: 10.1038/s41467-018-07305-8 (PMC6277390; doi:10.1038/s41467-018-07305-8)
Supplement: Supplementary file 1 — Supplementary Information [file 41467_2018_7305_MOESM1_ESM.pdf]

SUPPLEMENTARY INFORMATION

Microbiota-driven interleukin-17-producing cells and eosinophils synergize to accelerate multiple myeloma progression

Arianna Calcinotto, Arianna Brevi, Marta Chesi, Roberto Ferrarese, Laura Garcia Perez, Matteo Grioni, Shaji Kumar, Victoria M. Garbitt, Meaghen E. Sharik, Kimberly J Henderson, Giovanni Tonon, Michio Tomura, Yoshihiro Miwa, Enric Esplugues, Richard A. Flavell, Samuel Huber, Filippo Canducci, Vincent S Rajkumar, P Leif Bergsagel & Matteo Bellone

Supplementary Tables: 1

Supplementary Figures: 10

Supplementary Table 1. Patients' information

Pts	DX at Collection Sample	Collection Sample Date	MM DX Date	Group
1	SMM	3-Aug-04	25-Jun-10	Progression >3 years
2	SMM	11-Aug-05	25-Jun-10	Progression >3 years
3	SMM	28-Aug-06	25-Jun-10	Progression >3 years
4	SMM	20-Jul-07	24-Apr-14	Progression >3 years
5	SMM	23-Jul-09	24-Apr-14	Progression >3 years
6	SMM	8-Oct-07	6-Apr-11	Progression >3 years
7	SMM	27-Jan-06	10-Mar-10	Progression >3 years
8	SMM	29-Mar-05	14-Sep-10	Progression >3 years
9	SMM	13-Nov-06	4-Sep-15	Progression >3 years
10	SMM	24-Oct-05	10-Sep-10	Progression >3 years
11	SMM	18-Apr-06	10-Sep-10	Progression >3 years
12	SMM	28-Jun-06	1-Jun-10	Progression >3 years
13	SMM	16-Aug-07	25-Jun-10	Progression < 3 years
14	SMM	25-Sep-08	25-Jun-10	Progression < 3 years
15	SMM	19-May-06	24-Nov-08	Progression < 3 years
16	SMM	21-Aug-07	24-Nov-08	Progression < 3 years
17	SMM	3-Oct-08	4-Dec-09	Progression < 3 years
18	SMM	13-Dec-05	12-Mar-07	Progression < 3 years
19	SMM	7-Oct-08	14-Oct-09	Progression < 3 years
20	SMM	30-Jun-11	11-Jan-12	Progression < 3 years
21	SMM	26-Sep-07	27-Aug-08	Progression < 3 years
22	SMM	27-Nov-12	2-Mar-13	Progression < 3 years
23	SMM	2-Dec-10	6-Apr-11	Progression < 3 years
24	SMM	9-Jun-10	24-May-11	Progression < 3 years
25	SMM	2-Sep-08	10-Mar-10	Progression < 3 years
26	SMM	19-Jun-09	20-Jul-10	Progression < 3 years
27	MGUS	2-Feb-11	6-Feb-13	Progression < 3 years
28	SMM	3-Mar-09	29-Sep-09	Progression < 3 years
29	SMM	10-Oct-06	7-Jul-08	Progression < 3 years
30	SMM	15-Aug-05	31-Jul-07	Progression < 3 years
31	SMM	30-Nov-04	13-Apr-05	Progression < 3 years
32	SMM	18-Oct-07	16-Sep-08	Progression < 3 years
33	SMM	23-May-05	1-Dec-05	Progression < 3 years
34	SMM	24-Apr-07	8-Feb-10	Progression < 3 years
35	MGUS	31-Jul-09	19-Feb-10	Progression < 3 years
36	SMM	12-Jun-09	23-Dec-09	Progression < 3 years
37	MM	4-Dec-09	4-Dec-09	Before Treatment
38	MM	14-Oct-09	14-Oct-09	Before Treatment
39	MM	28-Apr-10	28-Apr-10	Before Treatment
40	MM	27-Aug-08	27-Aug-08	Before Treatment
41	MM	4-Apr-11	6-Apr-11	Before Treatment
42	MM	24-May-11	24-May-11	Before Treatment
43	MM	14-Sep-10	14-Sep-10	Before Treatment
44	MM	11-Mar-08	11-Mar-08	Before Treatment
45	MM	20-Jul-10	20-Jul-10	Before Treatment
46	MM	6-Feb-13	6-Feb-13	Before Treatment
47	MM	7-Jul-08	7-Jul-08	Before Treatment
48	MM	20-Sep-10	10-Sep-10	Before Treatment
49	MM	9-Feb-10	8-Feb-10	Before Treatment
50	MM	7-Aug-09	24-Nov-08	After Treatment
51	MM	12-Nov-07	12-Mar-07	After Treatment
52	MM	23-Sep-14	24-Apr-14	After Treatment
53	MM	18-May-12	11-Jan-12	After Treatment
54	MM	3-Jun-14	11-Jan-12	After Treatment
55	MM	3-Jan-12	6-Apr-11	After Treatment
56	MM	12-Jun-12	24-May-11	After Treatment
57	MM	16-Feb-15	24-May-11	After Treatment

58	MM	10-Nov-05	13-Apr-05	After Treatment
59	MM	14-Mar-11	10-Sep-10	After Treatment
60	MM	21-Mar-12	8-Feb-10	After Treatment

Abbreviations: Pts, patients; DX, Diagnosis.

Supplementary Figure 1

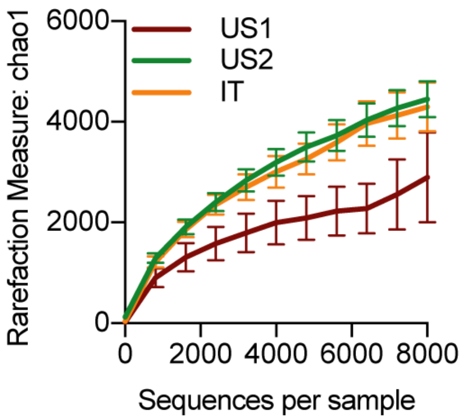

Microbiome analyses of stool samples from mice housed in the different animals facilities. CHAO1 index analysis of fecal microbiota from Vk*MYC and WT littermates housed in US1 (n=8 of biologically independent mice), US2 (n=16) and IT (n=8) animal facilities.

Supplementary Figure 2

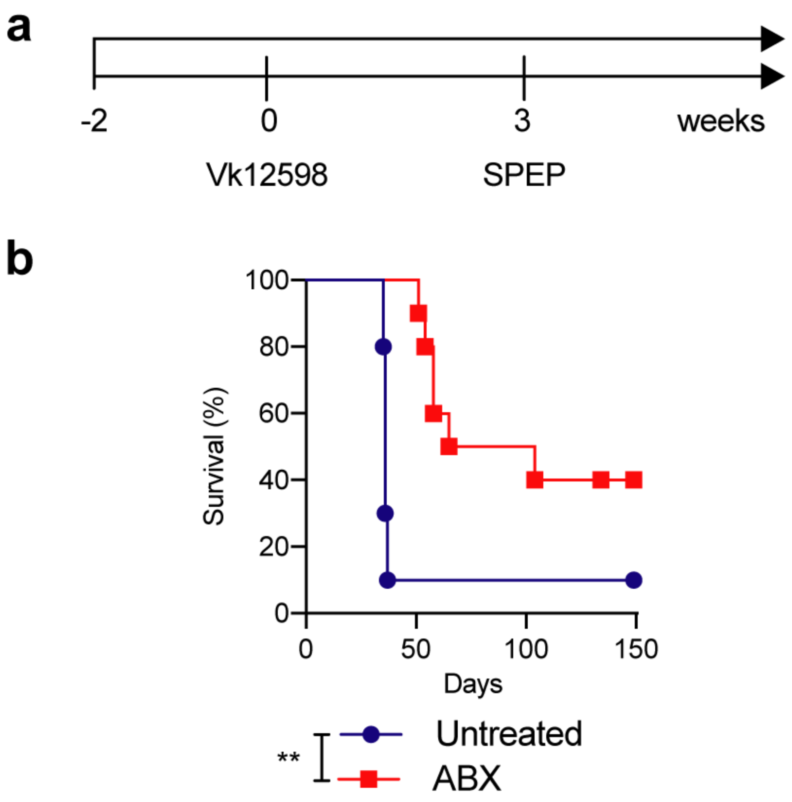

Antibiotic treatment improves the overall survival in t-Vk*MYC MM mice. a Schematic representation of the experiment reported in Fig. 1d. Mice were monitored for M-spike appearance as described in the Methods section. **b** Survival (Kaplan-Meier plot) of t-Vk*MYC MM mice maintained or not under antibiotic in drinking water (n=10/group) is reported. Long-rank (Mantel-Cox) test: *P =0.0027

Supplementary Figure 3

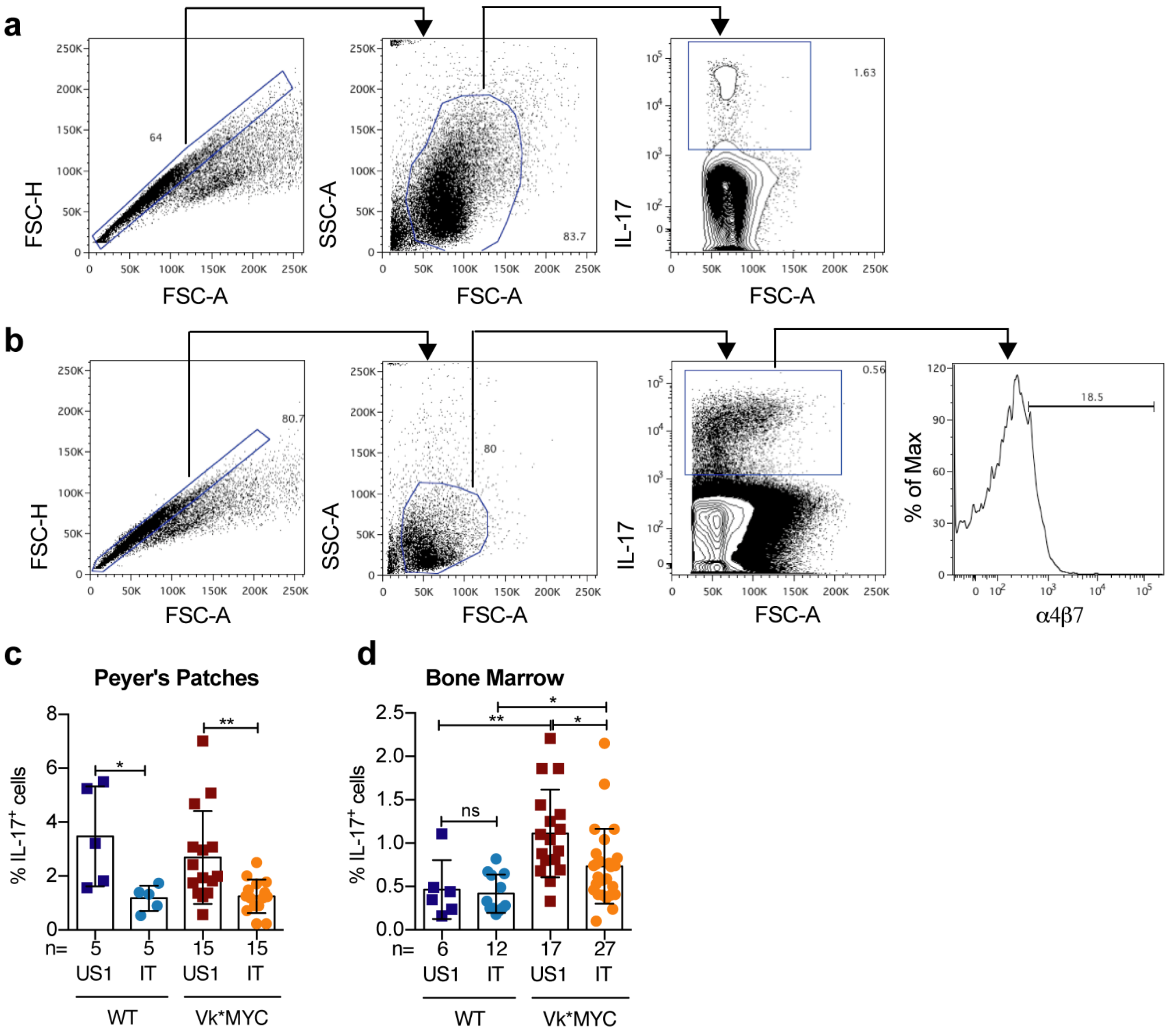

IL-17^{+} cells are enriched in the Peyer's patches and BM of Vk*MYC mice housed in the US1 animal facility. Gating strategy relative to IL-17^{+} cells in the Peyer's Patches (**a**) for the data reported in Fig. 1f, g, h and Suppl. Fig 2c, and in the BM (**b**) for the data reported in Fig. 1i, j, k, Fig. 2c, d, Fig. 6c, Suppl. Fig 2d, Suppl. Fig. 5a, b, c, d and Suppl. Fig. 10b. Frequency of IL-17^{+} cells in the Peyer's Patches (**c**) and BM (**d**) from Vk*MYC mice and sex- and age-matched WT littermates housed in US1 or in IT AF. Mean ± SD of three independent experiments. Unpaired *t* test: *P <0.05; **P <0.01; ***P <0.001.

Supplementary Figure 4

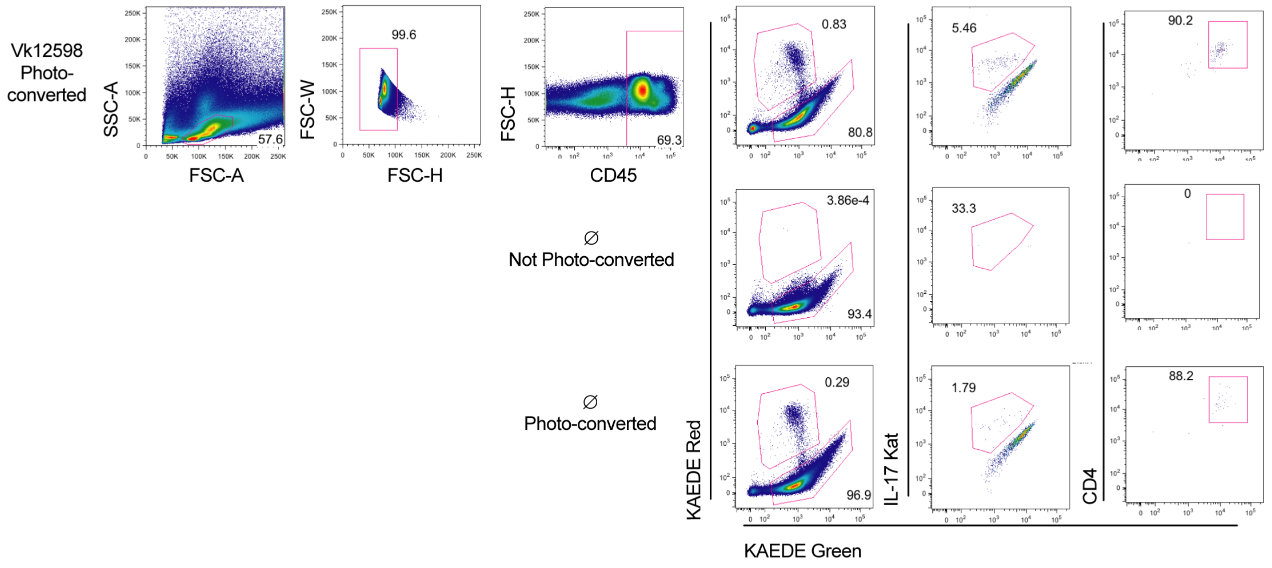

Gating strategy relative to the quantification of gut-experienced Th17 cells in photoconverted Kaede mice. Representative dot plots of BM cells from treated, photoconverted Kaede mice, untreated, not photoconverted Kaede mice and untreated, photoconverted Kaede mice (Fig. 1l and m). Right panels show frequency of IL-17A FP635^{+} cells within the KAEDE red positive and KAEDE red negative CD4^{+} T cells.

Supplementary Figure 5

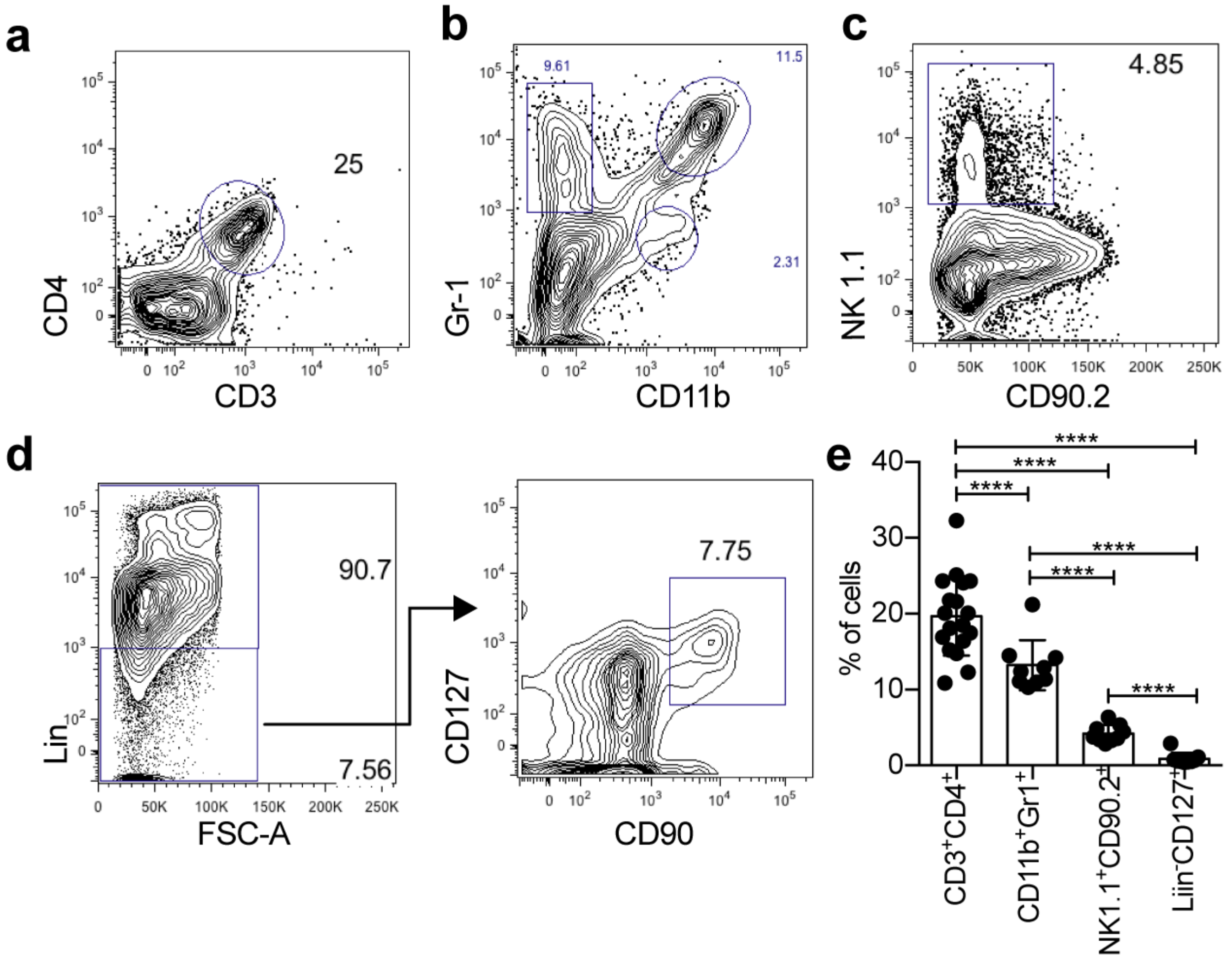

Characterization of IL-17$^+$ cells in the BM of Vk*MYC and WT mice. Representative plots of CD3$^+$CD4$^+$ (**a**), CD11b$^+$Gr-1$^+$ (**b**), NK1.1$^+$CD90.2$^-$ (**c**) and CD90$^+$CD127$^+$ cells (**d**) pre-gated on Lin- cells (lower left panel), all gated on IL-17$^+$ cells, in the BM of SPF Vk*MYC mice. Dead cells were excluded by live/dead staining. **e** Panels report the percentage of the different subsets. (CD3$^+$CD4$^+$ n=18; CD11b$^+$Gr-1$^+$ n=9; NK1.1$^+$CD90.2$^-$ n=9; Lin$^-$ CD127$^+$ n=9). Mean ± SD of three independent experiments. One-way Anova P < 0.0001. Unpaired *t* test: **P* <0.05; ***P* <0.01; ****P* <0.001; *****P* <0.0001.

Supplementary Figure 6

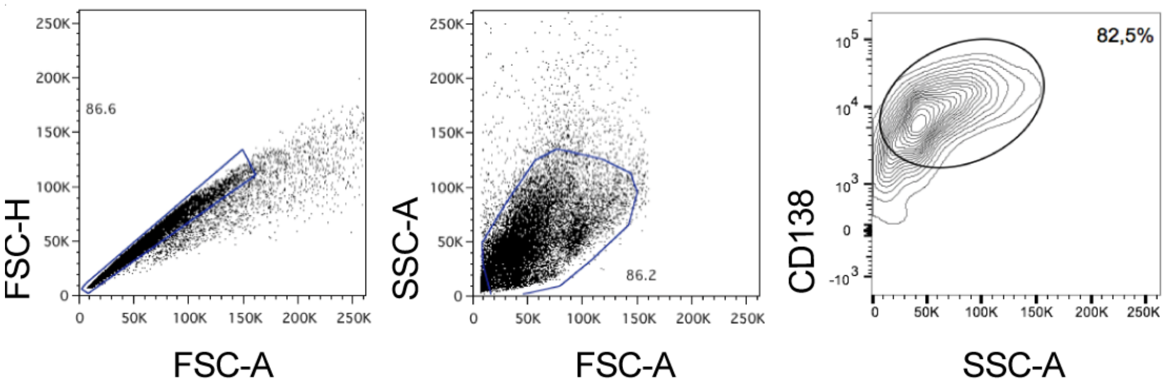

Gating strategy relative to BM-derived CD138$^+$ plasma cells. Gating strategy for the flow-cytometry analyses of CD138$^+$ plasma cells obtained from the BM of Vk*MYC mice, and reported in Fig. 3b, c, d.

Supplementary Figure 7

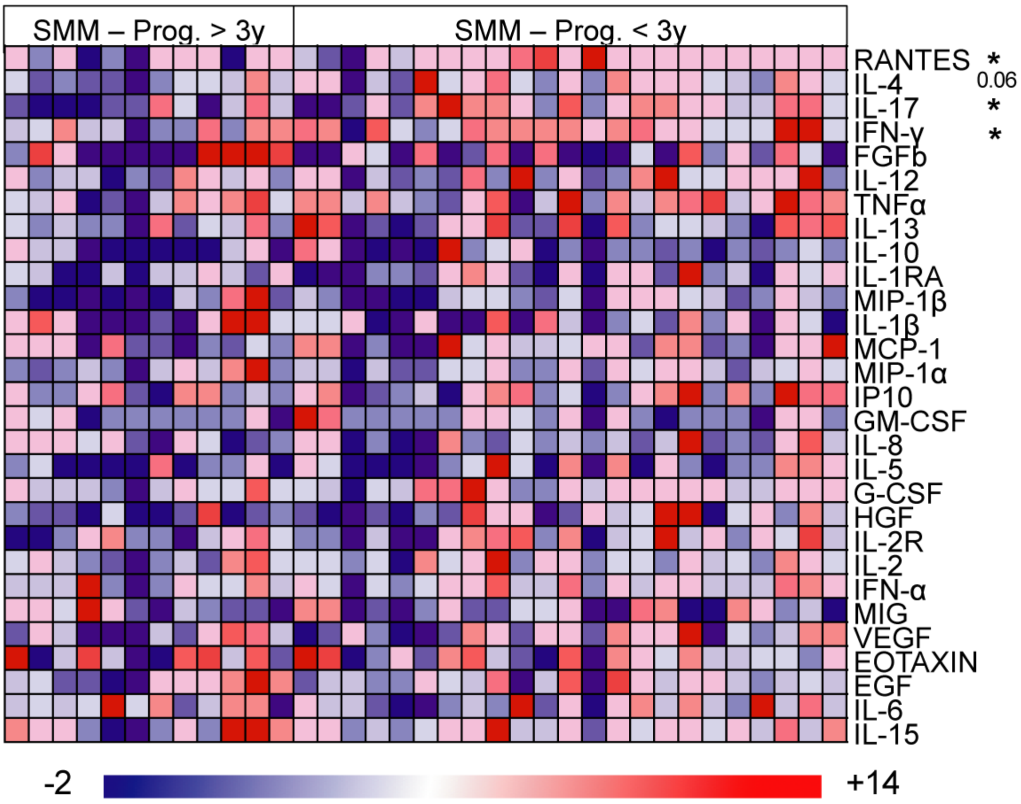

Quantification of cytokines and chemokines in the BM serum obtained from SMM patients. Cytokines and chemokines were quantified in the BM sera of SMM patients that progress to MM < 3 years, or > 3 years as described in the Materials and Methods section. The Heat map reports the relative expression (log base 2) of the indicated BM soluble factors ranked based on their differential expression between the two groups.

Supplementary Figure 8

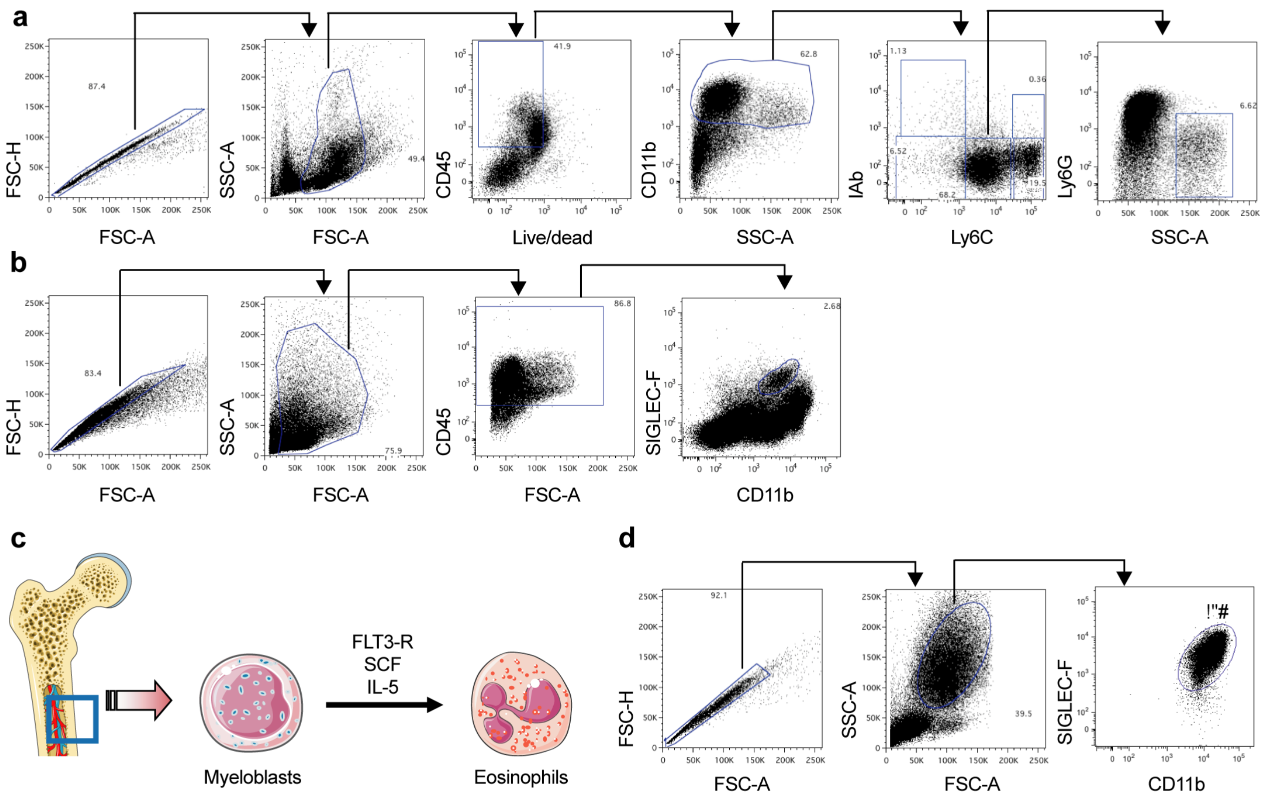

Quantification of BM eosinophils and their *in vitro* differentiation. a Gating strategy relative to the quantification of the frequency of eosinophils in the BM of Vk*MYC, Vk*MYC IL-17ko and t-Vk*MYC MM bearing mice in Fig. 5a and 6d. **b** Gating strategy relative to the quantification of IL-6-producing eosinophils from the BM of Vk*MYC, Vk*MYC IL-17ko and t-Vk*MYC MM bearing mice in Figs. 5b, c and d. **c** Graphic representation of the *in vitro* differentiation of BM derived eosinophils, (see Materials and Methods section for further information). **d** Representative gating strategy of BM derived eosinophils after 12 days of culture with the described cytokines, relative to data reported in Figs. 5e, f, g, h, and i.

Supplementary Figure 9

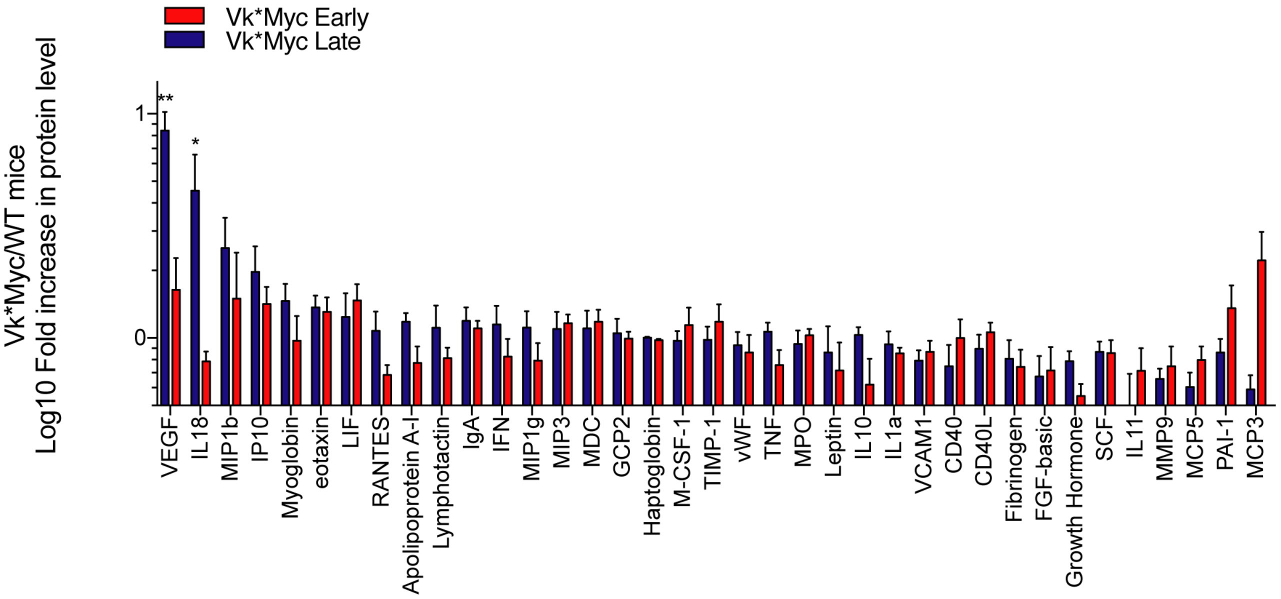

Quantification of cytokines and chemokines in the BM of Vk*MYC mice. Cytokines and chemokines were quantified in the BM of sex- and age-matched Early-MM (n=7), Late-MM Vk*MYC (n=5) and WT mice (n=5). The graph reports the relative amount (log base 10) of the indicated soluble factors normalized to the values obtained in the BM of WT mice. Unpaired t-test *P<0.05;**P<0.002.

Supplementary Figure 10

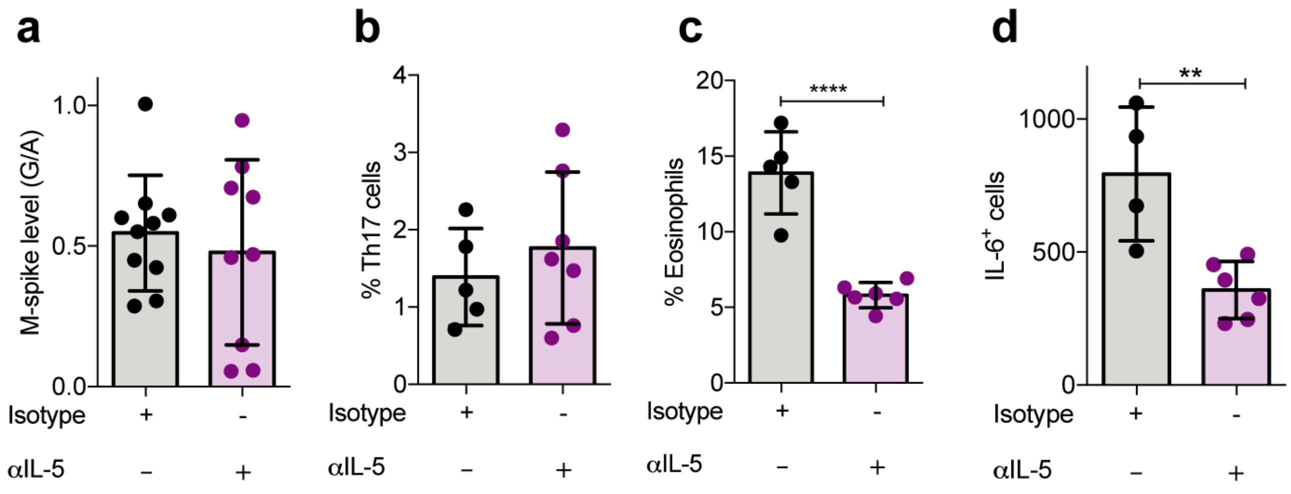

Treatment with anti-IL-5 antibodies does not impact disease progression in t-Vk*MYC MM mice. As indicated in the Material and Methods section, t-Vk*MYC MM mice were treated with anti-IL-5 antibodies (αIL-5), and euthanized for M-spike detection and BM analysis five weeks later. **a** M-spike levels are expressed as total gamma globulins/albumin ratio (G/A) in t-Vk*MYC MM mice within the indicated cohort at the time of sacrifice (Isotype n=10, αIL-5 n=9). **b** Frequency of Th17 cells in the indicated cohort (Isotype n=5, αIL-5 n=7). **c** Frequency of eosinophils in the indicated cohort (Isotype n=5, αIL-5 n=6). Each dot represents an individual mouse. Data are reported as mean ± SD. Unpaired *t* test: ****P <0.0001. **d** Number of IL-6^+ eosinophils in the indicated cohort (Isotype n=5, αIL-5 n=6). Unpaired *t* test: **P <0.01.
